# Enhanced volatile emissions and anti-herbivore functions mediated by the synergism between jasmonic acid and salicylic acid pathways in tea plants

**DOI:** 10.1093/hr/uhac144

**Published:** 2022-07-22

**Authors:** Long Jiao, Lei Bian, Zongxiu Luo, Zhaoqun Li, Chunli Xiu, Nanxia Fu, Xiaoming Cai, Zongmao Chen

**Affiliations:** Key Laboratory of Tea Biology and Resource Utilization, Ministry of Agriculture, Tea Research Institute, Chinese Academy of Agricultural Science, Hangzhou 310008, China; Key Laboratory of Tea Biology and Resource Utilization, Ministry of Agriculture, Tea Research Institute, Chinese Academy of Agricultural Science, Hangzhou 310008, China; Key Laboratory of Tea Biology and Resource Utilization, Ministry of Agriculture, Tea Research Institute, Chinese Academy of Agricultural Science, Hangzhou 310008, China; Key Laboratory of Tea Biology and Resource Utilization, Ministry of Agriculture, Tea Research Institute, Chinese Academy of Agricultural Science, Hangzhou 310008, China; Key Laboratory of Tea Biology and Resource Utilization, Ministry of Agriculture, Tea Research Institute, Chinese Academy of Agricultural Science, Hangzhou 310008, China; Key Laboratory of Tea Biology and Resource Utilization, Ministry of Agriculture, Tea Research Institute, Chinese Academy of Agricultural Science, Hangzhou 310008, China; Key Laboratory of Tea Biology and Resource Utilization, Ministry of Agriculture, Tea Research Institute, Chinese Academy of Agricultural Science, Hangzhou 310008, China; Key Laboratory of Tea Biology and Resource Utilization, Ministry of Agriculture, Tea Research Institute, Chinese Academy of Agricultural Science, Hangzhou 310008, China

## Abstract

The interaction between jasmonic acid (JA) and salicylic acid (SA) pathways, which affects plant stress resistance, is mainly considered to be antagonistic. Using an established theoretical model, we investigated how tea plant (*Camellia sinensis*) volatiles induced by exogenous elicitors of the JA and SA pathways are affected by the sequence of elicitor application, elicitor identity, and the applied concentrations. We also examined the effects of the volatiles mediated by the JA–SA synergistic interaction on the behaviors of a tea leaf-chewing herbivore (*Ectropis grisescens*) and its parasitic wasp (*Apanteles* sp.). The JA and SA pathway interactions were almost always reciprocally synergistic when the two pathways were elicited at different times, except at high JA elicitor concentrations. However, the JA pathway antagonized the SA pathway when they were elicited simultaneously. The elicitor identity affected the degree of JA–SA interaction. The volatiles induced by the JA pathway in the JA–SA reciprocal synergism treatments included up to 11 additional compounds and the total amount of volatiles was up to 7.9-fold higher. Similarly, the amount of emitted volatiles induced by the SA pathway in the reciprocal synergism treatments increased by up to 4.2-fold. Compared with the volatiles induced by either pathway, the enriched volatiles induced by the JA–SA reciprocal synergism similarly repelled *E. grisescens*, but attracted *Apanteles* sp. more strongly. Thus, non-simultaneous activation is important for optimizing the JA–SA reciprocal synergism. This reciprocal synergism enables plants to induce multifarious responses, leading to increased biotic stress resistance.

## Introduction

The jasmonic acid (JA) and salicylic acid (SA) pathways are two major phytohormone pathways related to anti-herbivore resistance in plants [1, 2]. Interactions between the JA and SA pathways allow a complex signaling network that modulates the plant metabolome and affects plant resistance to herbivores [3–7]. Currently, >80% of studies on JA–SA interactions have indicated that the interaction between the JA and SA pathways is reciprocally antagonistic [5]. Most of these studies focused on gene transcript levels and protein abundances in the upstream part of these pathways, with only a few focusing on the changes in downstream metabolites, which are particularly important for the ecological outcome of plant interactions with other organisms [5, 6]. This may be because it is difficult to accurately detect and quantify the many diverse metabolites that are produced as a result of the interaction between the JA and SA pathways [6, 7]. A previously reported theoretical model addressed this problem [7]. In this model, the compounds specifically produced after the activation of the JA or SA pathway are screened out from the metabolome as the features of the expression of the two pathways; these finite features can then be used to assess the JA–SA interaction [7].

The JA and SA pathways can be elicited specifically by hormone elicitors, such as JA/methyl jasmonate (MeJA) and SA/methyl salicylate (MeSA), respectively [8, 9]. Exogenous applications of JA and SA elicitors can change the profile of plant volatiles and quantitatively and qualitatively enrich the mixture of volatiles. These induced volatiles, which can repel herbivores and attract natural enemies of herbivores, play an important role in plant defense [10, 11]. There is substantial evidence showing that the composition of the volatiles induced by JA and SA elicitors and the associated ecological outcomes are completely different. The JA pathway-mediated volatiles are mainly composed of green leaf volatiles and terpenes that typically attract parasitic enemies of herbivores, whereas the SA pathway-mediated volatiles mainly consist of aromatic compounds that are primarily responsible for repelling ovipositing herbivores or attracting predators of herbivores [5, 9, 12–16]. Therefore, exogenous applications of these hormones could be used as part of a pest management strategy [17].

Tea (*Camellia sinensis*) is a perennial woody plant species and an important beverage crop worldwide. Previous studies showed that the exogenous application of JA and SA hormone elicitors can enhance several stress resistances in tea plants [18–21]. In this study, we determined the effects of the JA–SA interaction on the emitted volatiles as well as the anti-herbivore properties of tea plants. A theoretical model was established on the basis of the volatiles induced by these elicitors of the JA and SA pathways. Then, the effects of the sequence of the elicitor application, the elicitor identity (ID), and the elicitor concentration on the JA–SA pathway interaction were investigated using this model. We also evaluated the effect of volatiles mediated by the JA–SA interaction on the behaviors of the tea leaf-chewing herbivore *Ectropis grisescens* and the parasitic wasp of its larvae, *Apanteles* sp.

**Figure 1:**
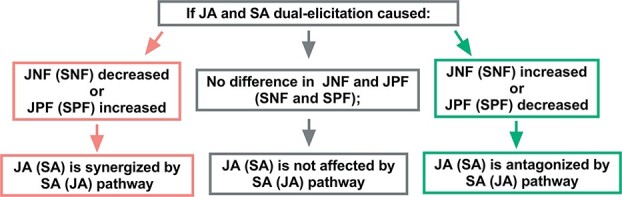
Theoretical model for studying the effect of the JA–SA interaction on induced tea plant volatiles. Compounds in the JNF and JPF groups were only induced by JA pathway elicitors; their emitted amount was respectively negatively and positively correlated with the concentration of JA pathway elicitors. Compounds in the SNF and SPF groups were only induced by SA pathway elicitors; their emitted amount was respectively negatively and positively correlated with the concentration of SA pathway elicitors.

## Results

### Establishment of a theoretical model for studying the jasmonic acid–salicylic acid interaction according to tea plant volatiles

A theoretical model was established for analyzing the effect of the JA–SA interaction on the emission of volatile compounds from tea plants (Fig. 1). The volatile compounds only induced by JA pathway elicitors were defined as JA pathway features, whereas those only induced by SA pathway elicitors were defined as SA pathway features. For JA pathway features, the compounds whose emitted amount was positively correlated with the concentration of JA pathway elicitors were defined as JA-positive features (JPFs), whereas those negatively correlated with the elicitor concentration were defined as JA-negative features (JNFs). The same method was used to classify SA pathway features as SA-positive features (SPFs) or SA-negative features (SNFs). The effect of the JA–SA interaction on the emission of volatiles was determined by comparing the JNF, JPF, SNF, and SPF emissions between JA and SA dual elicitation and the corresponding JA and SA single elicitation (Fig. 1). If the emission of JNFs (emission amount or number of compounds emitted) decreased or the emission of JPFs increased, the JA pathway was considered to be synergized by the SA pathway. In contrast, the JA pathway was considered to be antagonized by the SA pathway if the emission of JNFs increased or the emission of JPFs decreased. If there was no difference in JNF and JPF emissions, the SA pathway was considered to have no effect on the JA pathway. The effect of the JA pathway on the SA pathway was determined similarly.

**Figure 2:**
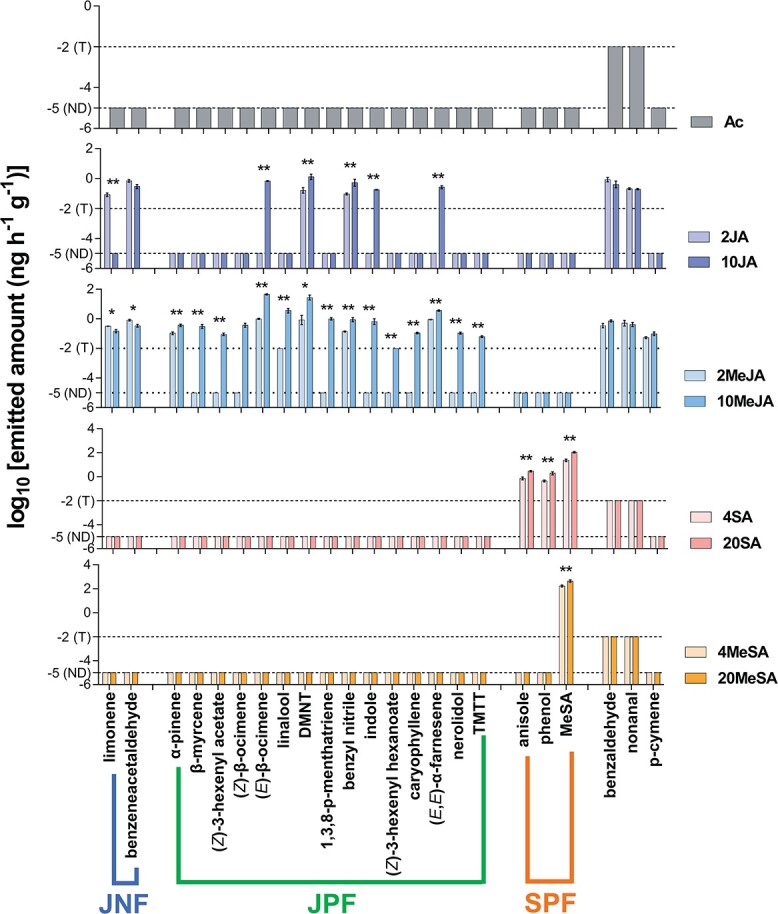
Feature definitions for JA and SA pathways in the theoretical model. Bar values represent the log-transformed amounts of emitted compounds (data are presented as mean ± standard error, *n* = 4). ND, not detected; T, <0.01 ng h^−1^ g^−1^ (signal-to-noise ratio = 3). Asterisks indicate significant differences in the emitted amount between two concentrations of the same elicitor (independent samples *t*-test; ^*^P < .05; ^**^P < .01). DMNT, (*E*)-4,8-dimethyl-1,3,7-nonatriene; TMTT, (*E*,*E*)-4,8,12-trimethyl-1,3,7,11-tridecatetraene; Ac, 2% acetone; 2JA, 2 mM JA; 10JA, 10 mM JA; 2MeJA, 2 mM MeJA; 10MeJA, 10 mM MeJA; 4SA, 4 mM SA; 20SA, 20 mM SA; 4MeSA, 4 mM MeSA; 10MeSA, 10 mM MeSA.

A total of 23 volatile compounds were induced by the JA and SA pathway elicitors in the single-elicitation treatments. Of these compounds, benzaldehyde and nonanal were emitted from control tea plants sprayed with acetone solution (Ac) and the emitted amounts increased significantly after the JA or MeJA treatment (*t*-test, *P* < .05; Fig. 2). The amount of MeSA emitted following the 4MeSA (4 mM MeSA) and 20MeSA (20 mM MeSA) treatments decreased gradually after spraying, dropping below the detection limit at 16 h and then increasing to a peak at 24 h (Supplementary Data Fig. S1). The emission of MeSA from tea plants was induced by spraying with MeSA (Fig. 2). The compounds induced by JA and SA pathway elicitors did not overlap, and those induced by the same pathway elicitors were similar (Fig. 2). Among the 23 compounds detected in this study, the 20 that were emitted in amounts proportional to the elicitor concentrations were defined as JNFs, JPFs, and SPFs according to the model. Benzeneacetaldehyde and limonene, whose emitted amount was negatively correlated with the concentration of JA or MeJA, were defined as JNFs. Similarly, 15 compounds, including (*Z*)-3-hexenyl acetate, (*E*)-β-ocimene, linalool, and indole, were defined as JPFs. Anisole, phenol, and MeSA were defined as SPFs (all defined compounds: *t*-test, *P* < .05; Fig. 2).

### Effects of sequence of elicitor application on jasmonic acid–salicylic acid interaction

**Figure 3:**
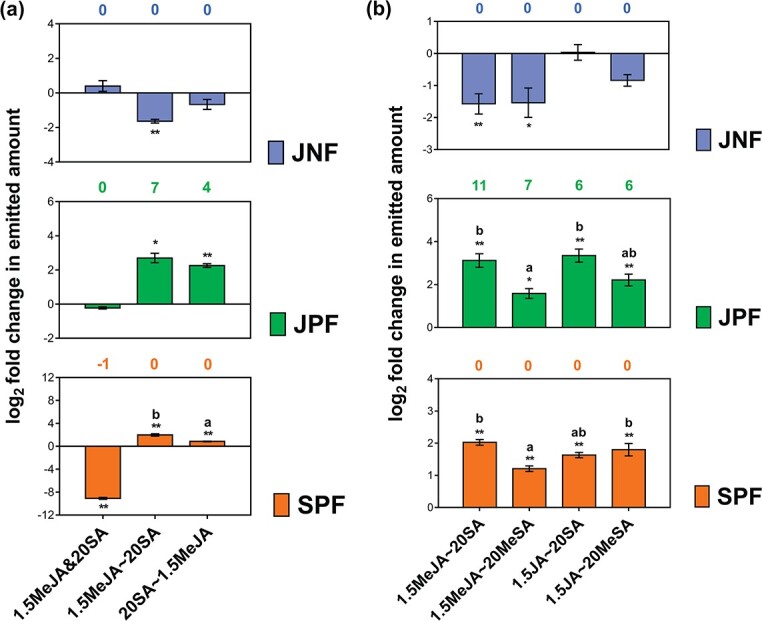
Effects of sequence of elicitor application and elicitor ID on volatile emissions mediated by the JA–SA interaction. **a** Effects of sequence of elicitor application on emissions of JNFs, JPFs, and SPFs. **b** Effects of elicitor ID on emissions of JNFs, JPFs, and SPFs. See Table 1 for treatment abbreviations. Bar values represent log_2_ fold changes in emitted amounts of JNFs, JPFs, and SPFs between the dual elicitation and the corresponding single elicitation (data are presented as mean ± standard error, *n* = 4). Asterisks indicate significant differences in the JNF, JPF, and SPF emitted amounts between dual elicitation and corresponding single elicitation (independent samples *t*-test, ^*^P < .05, ^**^P < .01). Different letters indicate significant differences in the log_2_ fold changes in the emitted amounts of JNFs, JPFs, and SPFs among different dual elicitations (independent samples *t*-test for two samples, *P* < .05; one-way ANOVA and *post hoc* Tukey’s test for more than two samples, *P* < .05). Numbers in blue, green, and orange respectively indicate the number of compounds in JNFs, JPFs, and SPFs after dual elicitation minus the number after the corresponding single elicitations.

The volatiles induced by the elicitors applied in different sequences are shown in Supplementary Data Fig. S2. Compared with the corresponding single elicitations, smaller amounts of JNFs, larger amounts of both JPFs and SPFs, and seven additional compounds in JPFs were emitted after pre-JA and post-SA elicitation (1.5MeJA ~ 20SA; ‘~’ indicates the two solutions were applied at different times) (JNF, JPF, and SPF amounts: *t*-test, all *P* < .05; Fig. 3a). The variations in JNFs, JPFs, and SPFs emitted after the pre-SA and post-JA elicitation (20SA ~ 1.5MeJA) were similar to those after the 1.5MeJA ~ 20SA treatment (JPF and SPF amounts: *t*-test, both *P* < .01; Fig. 3a). According to the model, the JA and SA pathways were reciprocally synergized under these conditions, and the synergism was stronger in the 1.5MeJA ~ 20SA treatment than in the 20SA ~ 1.5MeJA treatment (log_2_ fold change in SPF amount: *t*-test, *P* < .05; Fig. 3a). However, the number and amount of SPF emitted after the simultaneous MeJA and SA elicitation (1.5MeJA & 20SA; ‘&’ indicates simultaneous application of the different solutions) were lower than those after the corresponding single elicitations (SPF amount: *t*-test, *P* < .01; Fig. 3a). The number and amount of both JNFs and JPFs were similar in the 1.5MeJA & 20SA and 1.5MeJA treatments. Thus, the SA pathway was antagonized by the JA pathway when the two pathways were elicited simultaneously.

### Effects of exogenous elicitor identity on jasmonic acid–salicylic acid interaction

We analyzed the volatiles emitted after the application of different elicitors (Supplementary Data Fig. S3). Similar to the results described above, the JA and SA pathways were reciprocally synergized in the 1.5MeJA ~ 20SA, 1.5MeJA ~ 20MeSA, 1.5JA ~ 20SA, and 1.5JA ~ 20MeSA treatments (JNF, JPF, and SPF amounts: *t*-test, all *P* < .05, except for JNFs in 1.5JA ~ 20SA and 1.5JA ~ 20MeSA; Fig. 3b). The strongest JA–SA reciprocal synergism was in the 1.5MeJA ~ 20SA treatment (log_2_ fold change in JPF and SPF amounts: ANOVA, both *P* < .01; Fig. 3b).

### Effects of concentration of exogenous elicitors on jasmonic acid–salicylic acid interaction

**Figure 4:**
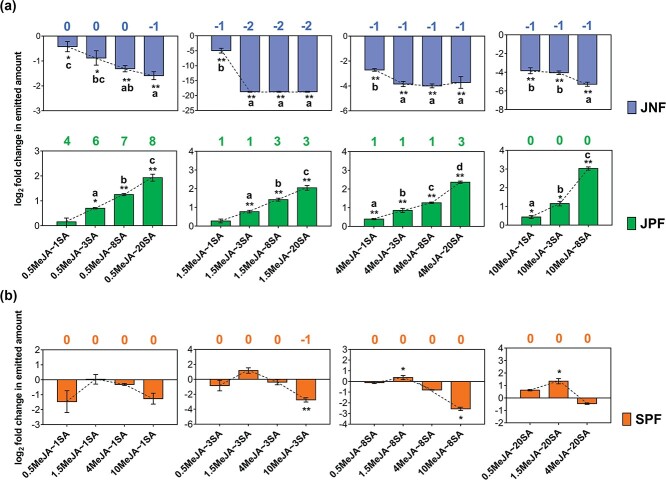
Effects of elicitor concentration on volatile emissions mediated by JA–SA interaction. **a** Effects of SA concentration on emission of JNFs and JPFs. **b** Effects of MeJA concentration on emission of SPFS. See Table 1 for treatment abbreviations. Bar values represent log_2_ fold changes in emitted amounts of JNFs, JPFs, and SPFsbetween dual elicitation and corresponding single elicitation (data are presented as mean ± standard error, *n* = 4). Mean values are linked by dashed lines. Asterisks indicate significant differences in the JNF, JPF, and SPF emitted amounts between dual elicitations and corresponding single elicitations (independent samples *t*-test, ^*^P < .05, ^**^P < .01). Different letters indicate significant differences in log_2_ fold changes in emitted amounts of JNFs, JPFs, and SPFs among different dual elicitations (one-way ANOVA and *post hoc* Tukey’s test, *P* < .05). Numbers in blue, green, and orange respectively indicate the number of compounds in JNFs, JPFs, and SPFs after dual elicitation minus the number after corresponding single elicitations.

We determined the effects of different elicitor concentrations on the emission of volatiles by tea plants (Supplementary Data Fig. S4). At all tested concentrations, the JA pathway was synergized by the SA pathway (JNF and JPF amounts: *t*-test, all *P* < .05, except for JPFs in 0.5MeJA ~ 1SA and 1.5MeJA ~ 1SA; Fig. 4a). Moreover, an increase in the SA concentration increased the synergistic effect on the JA pathway when the MeJA concentration was fixed (log_2_ fold change in JNF and JPF amounts: ANOVA, all *P* < .05; Fig. 4a). However, the effect of JA on the SA pathway was complicated and changed from synergistic to antagonistic as the MeJA concentration increased, especially when the SA concentration was 8 mM (SPF amounts in 10MeJA ~ 3SA, 1.5MeJA ~ 8SA, 10MeJA ~ 8SA, and 1.5MeJA ~ 20SA: *t*-test, all *P* < .05; Fig. 4b).

The results of the analyses of the sequence of elicitor application, exogenous elicitor ID, and the concentration of the exogenous elicitors showed that the interactions between the JA and SA pathways were mostly reciprocally synergistic when the pathways were elicited at different times, except at high MeJA concentrations. Compared with the effects of the single JA pathway elicitor treatment, there were 1–11 additional compounds and 4.2- to 7.9-fold more emitted volatiles induced by the JA pathway in the 1.5MeJA ~ 20SA treatment. Similarly, the total amount of emitted volatiles induced by the SA pathway in the 1.5MeJA ~ 20SA treatment was 2.9- to 4.2-fold higher than that induced by the single SA pathway elicitor treatment (Supplementary Data Figs S2–S4).

### Effect of jasmonic acid–salicylic acid reciprocal synergism on the preference of ovipositing *E. grisescens* moths and tropism of *Apanteles* sp. wasps

**Figure 5:**
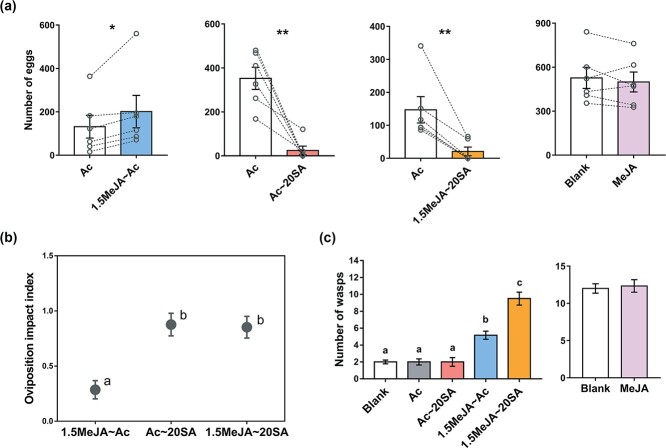
Effect of JA–SA reciprocal synergism on the behaviors of *E. grisescens* moths and *Apanteles* sp. wasps. Effect of JA–SA reciprocal synergism on the preference of ovipositing *E. grisescens* moths (**a**), the oviposition impact index of *E. grisescens* moths (**b**), and the tropism of *Apanteles* sp. wasps (**c**). Blank, empty odor source; MeJA, odor supplied by MeJA-immersed cotton ball. See Table 1 for other treatment abbreviations. Data are presented as mean ± standard error, *n* = 6. Asterisks in (**a**) indicate significant differences between treatments (paired samples *t*-test, ^*^P < .05, ^**^P < .01; small open circles represent the value of each replicate, and each pair is connected by dashed lines). Different letters in (**b**) and (**c**) indicate significant differences among treatments [one-way ANOVA and *post hoc* Tukey’s test in (**b**), generalized linear mixed model and *post hoc* Tukey’s test in (**c**), *P* < .01].

More eggs of *E. grisescens* moths were laid on the tea plants treated with 1.5MeJA ~ Ac than on the tea plants treated with Ac (*t*-test, *P* < .05; Fig. 5a). Significantly fewer eggs were laid on the tea plants treated with Ac ~ 20SA or 1.5MeJA ~ 20SA than on the tea plants treated with Ac (*t*-test, both *P* < .01; Fig. 5a). The oviposition impact indices in the Ac ~ 20SA and 1.5MeJA ~ 20SA treatments were similar and higher than that in the 1.5MeJA ~ Ac treatment (ANOVA, *P* < .01; Fig. 5b).

The *Apanteles* sp. wasps significantly preferred the tea plants treated with 1.5MeJA ~ 20SA and 1.5MeJA ~ Ac over the blank control tea plants and the tea plants treated with Ac and Ac ~ 20SA (generalized linear mixed model, *P* < .01; Fig. 5c). The tea plants treated with 1.5MeJA ~ 20SA were more attractive to wasps than the tea plants treated with 1.5MeJA ~ Ac. There was no significant difference in the number of wasps among the blank control and the Ac and Ac ~ 20SA treatments (Fig. 5c). The MeJA odor did not affect the behaviors of *E. grisescens* and *Apanteles* sp. (Fig. 5a and c).

## Discussion

In this study we did not detect the previously reported JA and SA common feature compounds and the JA–SA interaction feature compounds [7]. This difference might be because only volatile compounds induced by the elicitors and those whose abundance was affected by the concentration of a single elicitor were included in the model used to assess the effect of the JA–SA interaction in tea plants. The volatile compounds detected in this study represent a tiny fraction of plant metabolites. Moreover, the induced volatile compounds were highly specific to the two pathways because there were no overlapping compounds induced by the JA and SA elicitors (Fig. 2). This small number of pathway-specific analytical targets allowed convenient and accurate investigations of the JA–SA interaction. The expression of the pathways is reportedly correlated with the pathway elicitor concentration [22]. Therefore, investigating the effect of elicitor concentrations on the induced volatile emissions before analyzing the JA–SA interaction is important, otherwise the compounds induced by high concentrations of JA or SA elicitors might be included among the JA–SA interaction features.

In previous studies on the JA–SA interaction, the JA and SA pathways were mainly activated by exogenous chemical elicitors or biological elicitors (e.g. herbivore feeding and oviposition) [5]. Recent research indicated that the JA–SA interaction induced by tea geometrid feeding in tea plants may be antagonistic [23] or synergistic [24]. The differences in the results of these two studies might be mainly related to the difference in the sampling times. However, there is evidence that the activation of signaling pathways due to herbivore infestations is the result of mechanical damage as well as the elicitors in the oral secretions and egg-associated secretions [16, 25, 26]. Moreover, herbivore infestations can activate multiple signaling pathways (i.e. more than just the JA and SA pathways) [27, 28]. Therefore, only the results of studies in which the JA and SA pathways were elicited by exogenous chemical elicitors were compared with our results.

The JA–SA interaction was mainly designated as antagonistic in previous studies [5], which is inconsistent with our findings. Most of the earlier related research focused on the upstream interactions of the two pathways [5, 29]. However, the changes in downstream metabolites and ecological outcomes were often inconsistent with the interactions detected upstream [1, 5]. For example, although NPR1 in the SA pathway can suppress the expression of JA-regulated *PDF1.2* in *Arabidopsis thaliana*, SA does not affect JA-mediated resistance to *Alternaria brassicicola* [30, 31]. We surveyed studies on the downstream metabolites and ecological outcomes of JA–SA interactions and compared their findings with our results. Only 17 published papers involving 14 plant species were screened from a review [5] and searches of the Web of Science Core Collection database between 1 January 2000 and 31 December 2021 (Supplementary Data Table S1). These studies revealed that the interaction is antagonistic when the JA and SA pathways are elicited simultaneously [7, 11, 32–43] as well as when the two pathways are elicited at different times and the concentration of the previously applied elicitor is relatively high [11, 32, 44, 45]. In contrast, the interactions are synergistic when the two pathways are elicited at different times, with the previously applied elicitor used at low concentrations [45, 46]. For example, a 0.05 mM SA pretreatment can enhance the increase in the endogenous JA level in maize induced by 0.1 mM *N*-linolenoyl-glutamine, which is a JA pathway elicitor [45]. Therefore, the results of the 17 previous related studies are in accordance with our findings, which reflect the consistent effects of the sequence of the elicitor application and the elicitor concentrations on the JA–SA interaction. The sequence of the elicitor application and the elicitor concentrations affect the downstream metabolites as well as the upstream interactions [30, 32]; these effects can be considered as mutual antagonism or priming [47–50]. When elicited simultaneously, the JA and SA pathways may compete with each other for limited resources, leading to an antagonistic effect [1, 51]. Moreover, relatively low JA or SA elicitor concentrations can ‘prime’ plants to reserve genetic and metabolic resources for further activation. This allows plants to strengthen their response to the subsequently activated pathway [48, 52]. Approximately 70% of the studies on the JA–SA interaction have been conducted using simultaneous elicitation treatments [5]. We suggest that more studies should focus on the effects of the sequence of elicitor application and elicitor concentrations on JA–SA interaction at both the upstream and downstream levels.

Compared with the effects of the single elicitors of the JA and SA pathways, the effect of the JA–SA synergism on the emission of volatiles resulted in stronger resistance to *E. grisescens* (Fig. 5). Although the tea plant volatiles induced by 1.5MeJA ~ Ac and 1.5MeJA ~ 20SA contained attractants [53] (Supplementary Data Fig. S3), the ability of the volatiles induced by 1.5MeJA ~ 20SA to repel *E. grisescens* was equal to that of the volatiles induced by Ac ~ 20SA (Fig. 5a and b). This may be because the oviposition impact index of the repellents induced by Ac ~ 20SA was more than double the oviposition impact index of the attractants induced by 1.5MeJA ~ Ac (Fig. 5b). Among the volatiles induced by Ac ~ 20SA and 1.5MeJA ~ 20SA, the compound able to repel *E. grisescens* was probably MeSA because it accounted for 84–95% of the induced volatiles and is known to repel many lepidopterans [14, 54, 55]. In terms of wasps, the volatiles induced by 1.5MeJA ~ 20SA attracted wasps more than the volatiles induced by 1.5MeJA ~ Ac (Fig. 5c). This difference may be at least partly explained by the fact the 1.5MeJA ~ 20SA treatment increased the emission of some compounds, including (*Z*)-3-hexenyl acetate, (*Z*)-3-hexenyl hexanoate, and benzyl nitrile, which can elicit the antennal electrophysiological responses of *Apanteles* sp. wasps [56].

The reciprocal synergism between the JA and SA pathways is important for plant resistance to biotic stress. This synergism may increase the degree of resistance and/or expand the scope of resistance (compared with the resistance induced by a single pathway). For example, the JA and SA pathways in *Nicotiana glutinosa* work synergistically to mediate disease resistance. Compared with a single JA or SA treatment, JA and SA dual elicitation has greater inhibitory effects on the development of mosaic virus lesions on *N. glutinosa* leaves [46]. However, such effects have rarely been reported [5]. This is because in most previous studies the researchers simultaneously elicited the JA and SA pathways, leading to mutual suppression of the resistance induced by each pathway. In this study, the activation of the SA pathway enhanced the direct defense of tea plants by repelling ovipositing *E. grisescens*. The activation of the JA pathway enhanced the indirect defense of tea plants by attracting the parasitic wasp of *E. grisescens* larvae, but the induced volatiles also attracted *E. grisescens* females. However, in response to JA–SA reciprocal synergism, the tea plants exhibited two kinds of resistance by emitting volatiles at both the moth and larval stages of *E. grisescens*, rather than simply enhancing the direct or indirect defense levels. The JA pathway is responsible for defense against herbivores and necrotrophic pathogens, whereas the SA pathway is predominantly involved in defense against phloem sap-sucking insects and biotrophic pathogens [3, 5, 57]. Thus, whether the JA–SA reciprocal synergism can lead to simultaneous resistance to more biotic stresses, including herbivores and diseases, should be determined in future studies.

Tea is one of the most popular beverages worldwide, and its aroma is an important factor influencing tea quality [58]. The volatile compounds released by fresh tea leaves are responsible for tea aroma [59]. Previous studies revealed that the aroma quality of black and oolong teas can be improved by spraying the leaves of tea plants with MeJA, which results in increased emission of desirable aroma compounds, including terpenes and aromatic compounds [58, 60]. Thus, the changes to volatile emissions induced by the JA–SA synergism may be exploited to improve tea processing.

In conclusion, the tea plant volatiles induced by JA–SA interaction are affected by the sequence of elicitor treatment as well as by the ID and concentration of the elicitors. When the JA and SA pathways are not simultaneously elicited, they have a reciprocal synergistic interactive effect on volatile emissions. Because of this interaction, tea plants can simultaneously activate direct defense responses induced by the SA pathway and stronger indirect defense responses induced by the JA pathway against *E. grisescens.* Our research highlights the importance of considering the timing and dosage of elicitor applications when studying the plant JA–SA interaction. Moreover, the reciprocal synergism between the JA and SA pathways allows the plant to initiate multifarious responses that lead to stronger biotic stress resistance compared with that induced by eliciting a single pathway.

## Materials and methods

### Plants and insects

The tea plants (*Camellia sinensis* cultivar ‘Longjing 43’) used in this study were grown as previously described [21]. One-year-old tea plants were transplanted individually into plastic pots (25 cm height, 20 cm diameter) filled with potting soil, placed in a climate chamber [25 ± 2°C, 60–75% relative humidity (RH), 14 hours (05:00–19:00) light/10 hours (19:00–05:00) dark], watered to full soil capacity every week, and fertilized with rapeseed cake every 4 months. One year later, 2-year-old tea plants that were healthy, insect-free, and ~25 cm tall were used for the experiments.

*Ectropis grisescens* larvae were originally collected from the plantation of Shaoxing Royal Tea Village Co., Ltd. in Shaoxing, China. The larvae were reared on fresh tea plant shoots in a climate chamber [25 ± 2°C, 60–75% RH, 14 hours (20:00–10:00) light/10 hours (10:00–20:00) dark]. Male and female pupae were kept separately in cages. After eclosion, male and female moths were separately fed with a honey solution (10% in water) for 1 day. Then, one female and two male moths (1 day old) were confined in clean plastic containers (9 cm height, 8 cm diameter) for 1 day to obtain mated females. These 2-day-old mated females that had no contact with plant materials and no oviposition experience were used for bioassays.

**Table 1:** Treatments to test the effects of the sequence of the elicitor application, elicitor ID, and elicitor concentration on JA–SA interaction in tea plants

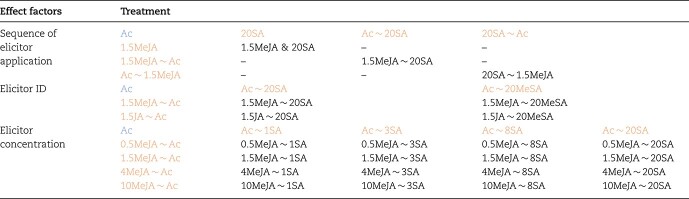

Treatments in black are dual elicitations of JA and SA pathways. Treatments in orange are the corresponding single elicitations of the JA (SA) pathway for the dual elicitations in that row (column). Treatments in blue are the controls. ‘–’ indicates no treatment. Numbers represent the elicitor solution concentrations (in mM). Ac, MeJA, JA, MeSA, and SA respectively represent 2% acetone, methyl jasmonate, jasmonic acid, methyl salicylate, and salicylic acid solutions. ‘&’ and ‘~’ indicate two solutions were applied simultaneously and at different times, respectively. Solutions before and after ‘~’ are the pre-spray and post-spray solutions, respectively.

Wasps (*Apanteles* sp.) that parasitize *E*. *grisescens* larvae were originally collected from the plantation of Shaoxing Royal Tea Village Co., Ltd. and then reared on *E*. *grisescens* larvae in a climate chamber [25 ± 2°C, 60–75% RH, 14 hours (06:30–20:30) light/10 hours (20:30–6:30) dark]. *Apanteles* sp. cocoons were collected and placed in cages. Newly emerged wasps (one female and two males) were immediately transferred to a centrifuge tube (10 mL), fed with a 10% honey solution, and kept for 1 day for mating. The 1-day-old mated females were used for bioassays. These wasps contacted neither plant materials nor caterpillars and had no oviposition experience.

### Chemicals and preparation of elicitor solutions

We used four exogenous elicitors of JA and SA pathways in this study. JA (≥85% purity; TCI, Tokyo, Japan) and MeJA (≥95% purity; Sigma–Aldrich, Saint Louis, MO, USA) were used to elicit the JA pathway, whereas SA (≥99%; J&K, Beijing, China) and MeSA (≥99%; J&K) were used to elicit the SA pathway. All four elicitors were first dissolved in acetone and then diluted to the required concentrations with tap water. A solution comprising MeJA and SA was prepared by diluting the mixture of these two elicitors first with acetone and then with tap water. The final acetone concentration in all solutions was 2% (v/v). The elicitor concentrations were selected on the basis of the results of preliminary experiments and a previous study [21]. The (*E*)-4,8-dimethyl-1,3,7-nonatriene (DMNT), (*E*,*E*)-α-farnesene, and (*E*,*E*)-4,8,12-trimethyl-1,3,7,11-tridecatetraene (TMTT) standards were synthesized by Laviana Corp. (Taizhou, Jiangsu, China). The standards of the other volatile compounds and the internal standard (decanoic acid ethyl ester) were obtained from Sigma–Aldrich.

### Tea plant treatments

Four experiments were conducted to study the effect of the JA–SA interaction on tea plant volatile emissions. In experiment 1, we investigated the volatiles induced by single elicitors of the JA and SA pathways. In experiments 2, 3, and 4 we investigated the effects of the sequence of the elicitor application, the elicitor ID, and the elicitor concentration, respectively, on the tea plant volatiles induced by the JA–SA interaction (Table 1).

In experiment 1, the tea plants were sprayed with one of two concentrations of JA, MeJA, SA, and MeSA solutions, respectively, at 12:00. The concentrations of both JA and MeJA were 2 and 10 mM, whereas the concentrations of both SA and MeSA were 4 and 20 mM. Tea plants were sprayed with 2% acetone (Ac) at 12.00 as the control. Additionally, to verify whether MeSA emission can be induced by spraying with a MeSA solution, the emission dynamics of MeSA from the treated tea plants were investigated (Supplementary Data Fig. S1).

In experiment 2, the tea plants were sprayed with a solution containing 1.5 mM MeJA and 20 mM SA at 12.00 (simultaneous elicitation of JA and SA pathways, 1.5MeJA & 20SA). Alternatively, they were pre-sprayed with 1.5 mM MeJA at 12:00 and post-sprayed with 20 mM SA after 12 hours (1.5MeJA ~ 20SA) or they were pre-sprayed with 20 mM SA at 12:00 and post-sprayed with 1.5 mM MeJA after 12 hours (20SA ~ 1.5MeJA).

In experiment 3, the tea plants were pre-sprayed with 1.5 mM JA pathway elicitor (JA or MeJA) at 12:00 and post-sprayed with 20 mM SA pathway elicitor (SA or MeSA) after 12 hours.

In experiment 4, the tea plants were pre-sprayed with MeJA (0.5, 1.5, 4, or 10 mM) at 12:00 and post-sprayed with SA (1, 3, 8, or 20 mM) after 12 hours. Because of phytotoxic effects, we did not include the treatment involving a pre-spray with 10 mM MeJA and a post-spray with 20 mM SA.

In experiments 2, 3, and 4, the tea plants were sprayed with a single elicitor (of the JA or SA pathway) and Ac as the corresponding single elicitation and control, respectively (Table 1). The corresponding single elicitation was conducted at the same time as the dual elicitation. All control tea plants in experiments 2–4 were only sprayed at 12:00. The spraying time was selected on the basis of the results of preliminary experiments. At each spraying time, individual tea plants were sprayed with 50 mL solution until the solution dripped from the leaves. Each treated plant was immediately covered with transparent plastic material (60 × 60 × 60 cm) and then transferred to a climate chamber [air-ventilated, 120 m^2^, 25 ± 2°C, 60–75% RH, 14 hours (05:00–19:00) light/10 hours (19:00–05:00) dark], where they were incubated until the volatiles were collected. All treatments were replicated using four individual plants.

### Collection and analysis of tea plant volatiles

Tea plant volatiles were collected using a dynamic headspace sampling system as previously described [21]. Purified air entered the glass cylinder (30 cm height, 25 cm diameter) containing the above-ground parts of the tea plant via Teflon tubes at a rate of 1200 mL min^−1^. The air was pulled out through a glass tubular trap filled with 35 mg Super-Q adsorbent (80–100 mesh; Alltech Associates, Deerfield, IL, USA) at a rate of 400 mL min^−1^. Volatiles from the tea plants were collected for 1 hour at 12:00 on the second day after the first spraying. To analyze the emission dynamics of MeSA, the volatiles were collected for 1 hour at 4, 8, 12, 16, 20, 24, 28, 32, 36, 40, 44, and 48 hours after spraying; the airway remained open in the collection system throughout the collection period. After the volatiles were collected, all of the leaves on each plant were removed and weighed to calculate the amount of volatiles emitted per unit biomass.

The same four climate chambers [air-ventilated, 25 ± 2°C, 60–75% RH, 14 hours (05:00–19:00) light/10 hours (19:00–05:00) dark] were used for collecting volatiles, with each chamber accommodating seven tea plants. For each treatment, volatiles were collected from four replicates in different chambers at the same time. In each chamber, volatiles from an untreated tea plant were also collected and analyzed as the system blank. The 1.5MeJA ~ 20SA treatment in experiments 2–4 was used to check the stability of the collection system. The volatile collection experiments were completed within 70 days.

The volatile compounds were analyzed according to previously described methods [21]. Briefly, volatile compounds were extracted from the traps using 500 μL methylene dichloride, after which 50 ng decanoic acid ethyl ester was added to the extract as an internal standard. Samples were analyzed using a gas chromatography–mass spectrometry system (GC 7890B-MSD 5977B; Agilent, Santa Clara, CA, USA). The gas chromatograph was equipped with an HP-5MS UI column (30 m × 0.25 mm inner diameter, 0.25 μm film thickness; J&W, Folsom, CA, USA). Samples were injected using a splitless injection technique at 200°C. The helium gas flow rate was 1.2 mL min^−1^. The oven temperature was maintained at 45°C for 2 min, increased by 5°C min^−1^ to 210°C, increased by 25°C min^−1^ to 240°C, and then maintained for 10 minutes. Ionization was achieved by electron impact at 70 eV and 230°C. Compounds were identified by comparing their mass spectra and retention times with those of authentic standards. The amounts of individual volatile compounds were calculated by comparing their peak areas with that of the internal standard.

### Insect bioassays

The effects of the JA–SA synergism on the oviposition preference of *E. grisescens* moths and the tropism of *Apanteles* sp. wasps were investigated. Tea plants treated with 1.5MeJA ~ Ac, Ac ~ 20SA, 1.5MeJA ~ 20SA, and Ac as described above were used as the odor sources.

A two-choice H-shaped olfactometer was used to evaluate the oviposition preference of *E*. *grisescens* moths. In this olfactometer, two acrylic cages (60 × 60 × 60 cm) were used as the odor source cages, and four tea plants that underwent the same treatment were placed in a cage. The cages containing the two odor sources were connected by an acrylic tube (110 cm long, 24 cm inner diameter) with a hole (3 cm diameter) in the middle for releasing the moths. The two ends of the tube were covered with nylon mesh (16 mesh), and Z-shaped paper bars were placed near the nylon mesh for oviposition. Four pairs of odors were tested: MeJA versus blank, Ac versus 1.5MeJA ~ Ac, Ac versus Ac ~ 20SA, and Ac versus 1.5MeJA ~ 20SA, with each pair tested six times. The MeJA versus blank comparison was used to investigate the effect of the MeJA odor on oviposition preference. The MeJA odor was produced by applying 1 mL MeJA standard to a cotton ball. The blank was an empty odor source cage. For each test, six mated females were released into the olfactometer 22 hours after the first spraying of the tea plant (at 10:00). The eggs laid on the Z-shaped paper bars and nylon mesh were counted 10 hours after releasing the moths. One replicate of the four comparison tests was analyzed at the same time in a darkroom (air-ventilated, 25 ± 2°C, 60–75% RH). Olfactometers were separated by at least 5 m. The direction of the olfactometer and the site of the odor source were completely random in each test. The olfactometer was cleaned with distilled water and dried under an airflow after each test.

The tropism of *Apanteles* sp. wasps was tested using a six-arm olfactometer under controlled conditions (25 ± 2°C, 60–75% RH) as previously described [61]. The apparatus had three shelves: the top shelf housed the olfactometer, the middle shelf was the insect release site, and the bottom shelf held the odor sources. Pure humidified air was pushed into the lower part of each odor source vessel (glass, 65 cm height, 35 cm diameter) at 600 mL min^−1^. Then, the air from each vessel was carried via a Teflon tube to an arm of the olfactometer. All six airflows came together in a central glass chamber, in which the wasps were released and showed a preference for an odor by walking into one of the arms. One LED bulb (25 W) at the center of the bottom shelf provided the light source for the tea plants. Another LED bulb (25 W) was positioned 60 cm above the central chamber. Six wasps were released into the olfactometer as a group and were allowed to choose among the six odor sources: Ac, Ac ~ 20SA, 1.5MeJA ~ Ac, 1.5MeJA ~ 20SA, and two empty odor source vessels as the blank control. After 30 min or as soon as all of the wasps had made a choice, the number of wasps in each trapping bulb was recorded. The wasps were removed before a new group was released. The tropism of the wasps to MeJA was also investigated, with the tested odors provided by three MeJA and three empty odor source vessels. The MeJA odor was produced by applying 0.2 mL MeJA standard to a cotton ball. For each olfactometer experiment, one replicate comprising five groups of wasps was tested from 10:00 to 14:00 on a given day. Each experiment was replicated six times within 10 consecutive days. The position of the tested odor source was randomly assigned on each experimental day. At the end of each day, all olfactometer parts were washed with water and acetone and then dried in an oven at 120°C.

### Statistical analyses

Unless otherwise stated, data were analyzed using SPSS 26 software (IBM, Armonk, NY, USA). Data used for *t*-tests and ANOVA were log-transformed to normalize their distribution and homogenize the variances.

For the volatile compound analyses, the independent samples *t*-test was used to analyze the differences in the amounts of benzaldehyde and nonanal emitted between the Ac treatment and the JA pathway elicitor treatment as well as the differences in the amounts of volatile compounds emitted between two concentrations of an elicitor in experiment 1. In experiments 2, 3, and 4 the differences in the amounts of JNFs, JPFs, and SPFs emitted between the dual elicitation and the corresponding single elicitation were analyzed by the independent samples *t*-test [62]. The log_2_ fold change in the amount and the difference in the number of compounds emitted were determined to compare JNF, JPF, and SPF emissions between the dual elicitation and the corresponding single elicitation. The log_2_ fold change in the amounts of JNFs, JPFs, and SPFs was calculated by dividing the amount emitted after the dual elicitation by the amount emitted after the corresponding single elicitation, followed by a log-transformation of the data. The difference in the number of JNF, JPF, and SPF compounds was calculated by subtracting the number of compounds produced after a single elicitation from the number of compounds produced after a dual elicitation. To quantify the JA–SA interaction among dual-elicitation treatments, significant log_2_ fold changes in the amount of JNFs, JPFs, and SPFs emitted were compared using the independent samples *t*-test (two samples) or a one-way ANOVA with *post hoc* Tukey’s test (more than two samples) [63].

For the insect bioassays, the effect of the tested odors on *E*. *grisescens* oviposition was determined by calculating the oviposition impact index using the following equation:

$$ \begin{align*}
\mid\! T-C\! \mid /(T+C),\end{align*}$$

where *T* is the number of eggs on the tea plant treated with elicitors and *C* is the number of eggs on the tea plant treated with Ac. The paired samples *t*-test was used to analyze the differences in the number of moth eggs between the two sides of the H-shaped olfactometer [64]. A one-way ANOVA and Tukey’s *post hoc* test were used to analyze the differences in the oviposition impact index among different treatments. The tropism of wasps to different odors in a six-arm olfactometer was examined using a generalized linear mixed model with Poisson distribution of error. Tukey’s *post hoc* test was used for multiple comparisons. The model was fitted according to a maximum quasi-likelihood estimation in the software package R and was checked with the overdispersion test to estimate the residual deviation of the freedom factor [65].

## Acknowledgements

This work was supported by the Modern Agricultural Industry Technology System (CARS-19), the National Key Research & Development Plan (2016YFD0200900), and the Key Research and Development Program of Zhejiang Province (2019C02033). We thank Liwen Bianji (Edanz) (www.liwenbianji.cn) for editing the English text of a draft of this manuscript.

## Author contributions

Z.C., X.C., and L.J. conceived the study. L.J. performed the experiments and analyzed the data. L.J. and X.C. wrote the manuscript. All authors contributed critically to the revision of the manuscript and gave final approval for publication.

## Data availability

All data needed to evaluate the conclusions in this paper are presented in the paper and the supplementary information. Additional data related to this paper may be requested from the authors.

## Conflict of interest

The authors declare no competing interests.

## Supplementary data

Supplementary data is available at *Horticulture Research* online.

## Supplementary Material

Web_Material_uhac144
