# Supplementary material for: Enhanced volatile emissions and anti-herbivore functions mediated by the synergism between jasmonic acid and salicylic acid pathways in tea plants
Source: Hortic Res. 2022 Jul 22;9:uhac144. doi: 10.1093/hr/uhac144 (PMC9463459; doi:10.1093/hr/uhac144)
Supplement: Web_Material_uhac144 [file web_material_uhac144.doc]

**Supplementary information**


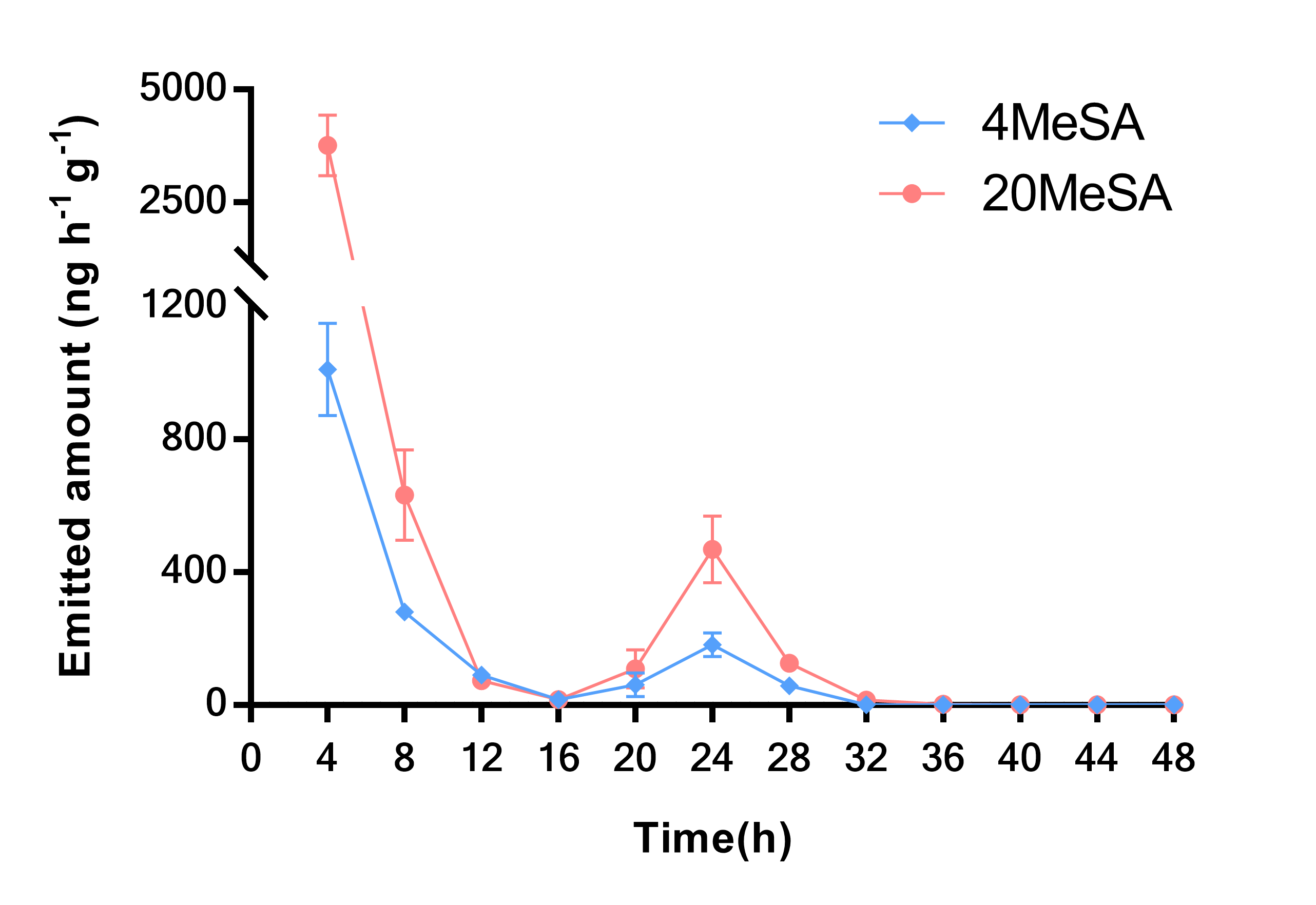


**Figure S1.** Emission dynamics of MeSA from tea plants sprayed with MeSA at two concentrations. Data are means ± SE, *n* = 4. 4MeSA, 4 mM MeSA. 20MeSA, 20 mM MeSA.


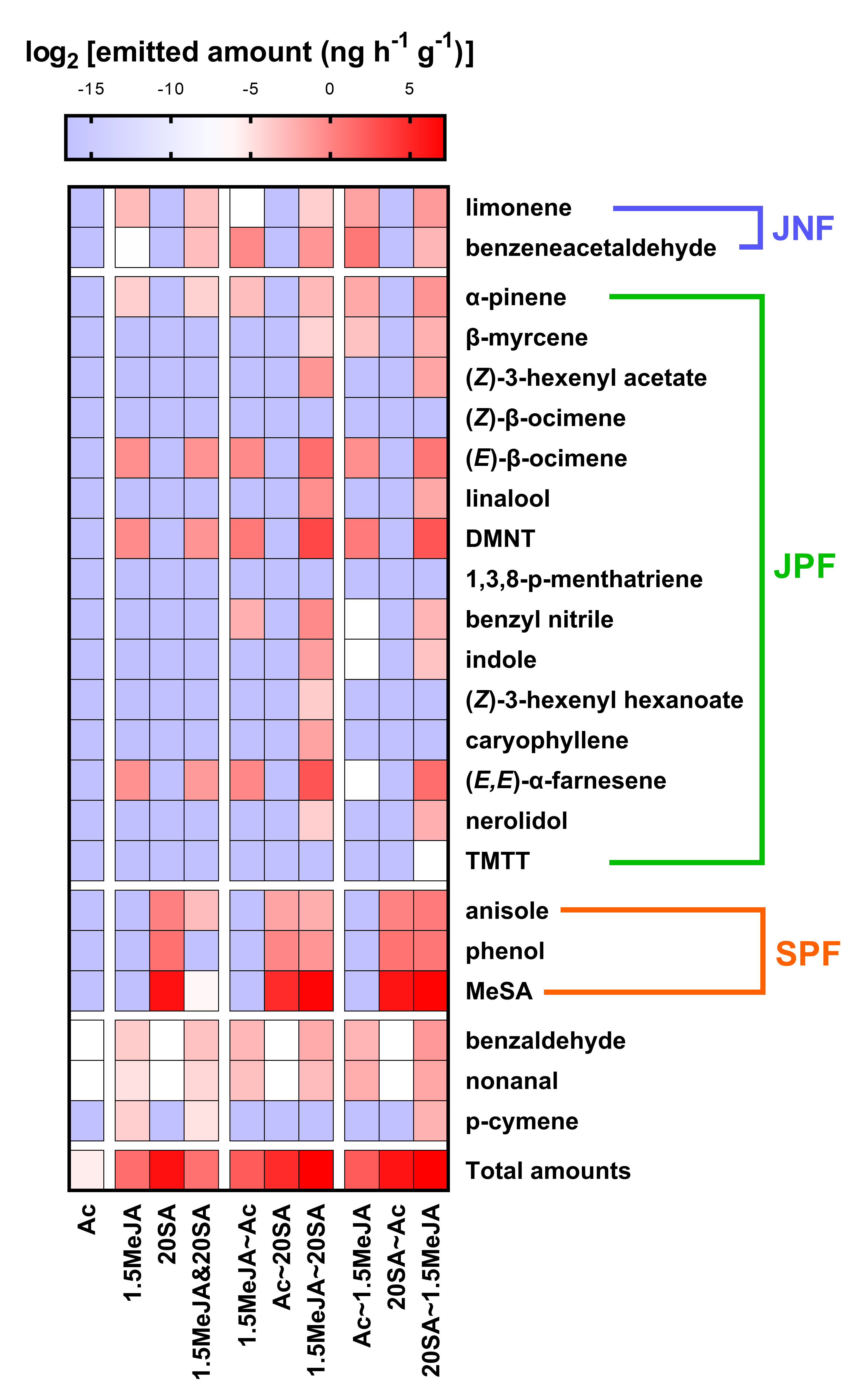


**Figure S2.** Tea plant volatiles induced by methyl jasmonate and salicylic acid applied at different sequences. JNF, JA negative features. JPF, JA positive features. SPF, SA positive features. DMNT, (*E*)-4,8-dimethyl-1,3,7-nonatriene. TMTT, (*E*,*E*)-4,8,12-trimethyl-1,3,7,11-tridecatetraene. MeSA, methyl salicylate. For treatment abbreviations, see Table 1. Data are given as log_2_ (mean emitted amount), *n* = 4. Blue and white cells represent, respectively, that compound was not detected or detected at less than 0.01 ng h^-1^ g^-1^ (signal to noise ratio = 3).


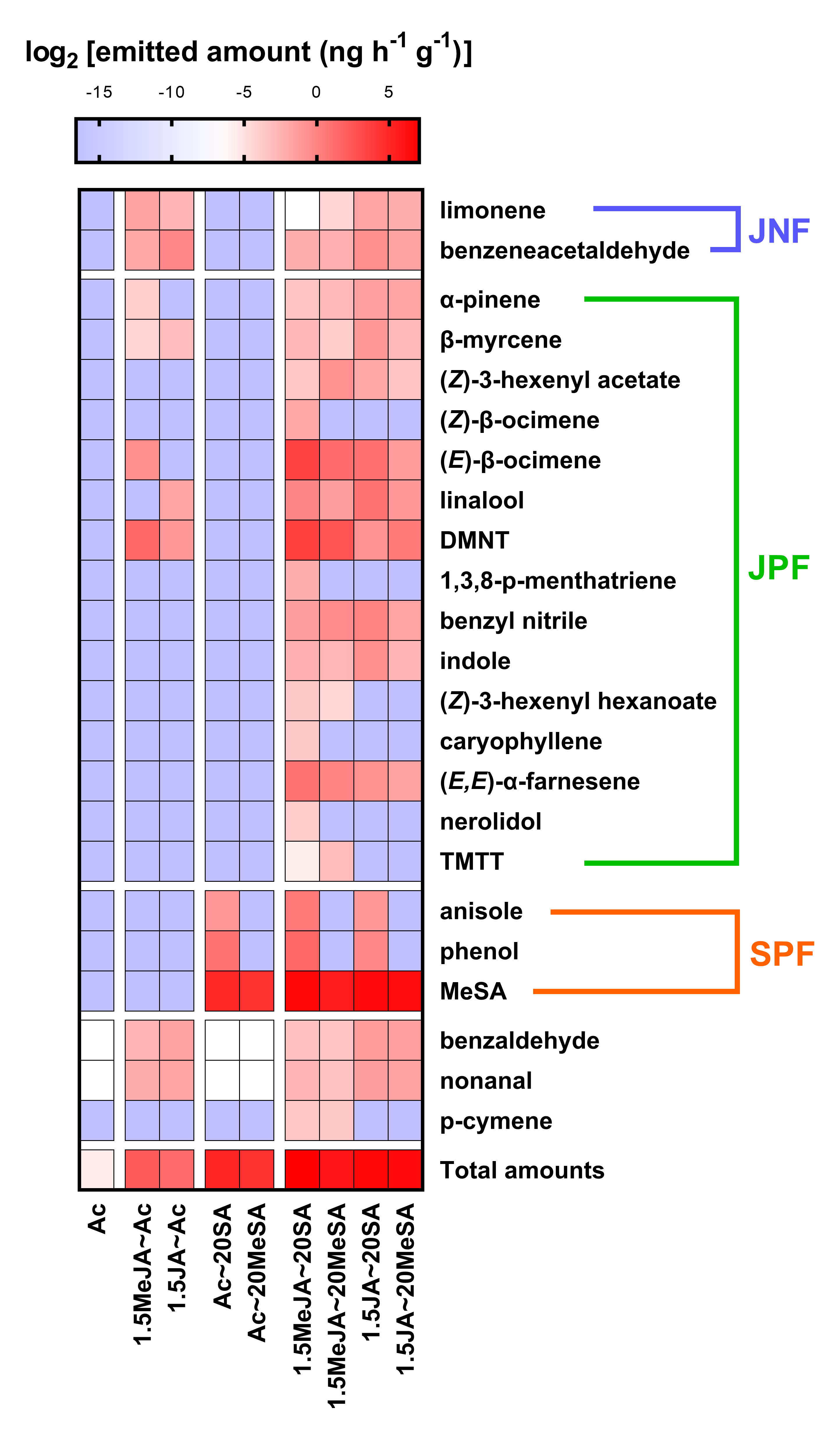


**Figure S3.** Tea plant volatiles induced by different jasmonic acid (JA) and salicylic acid (SA) pathway elicitors. JNF, JA negative features. JPF, JA positive features. SPF, SA positive features. DMNT, (*E*)-4,8-dimethyl-1,3,7-nonatriene. TMTT, (*E*,*E*)-4,8,12-trimethyl-1,3,7,11-tridecatetraene. MeSA, methyl salicylate. See Table 1 for treatment abbreviations. Data are given as log_2_ (mean emitted amount), *n* = 4. Blue and white cells represent, respectively, that compound was not detected or detected at less than 0.01 ng h^-1^ g^-1^ (signal to noise ratio = 3).


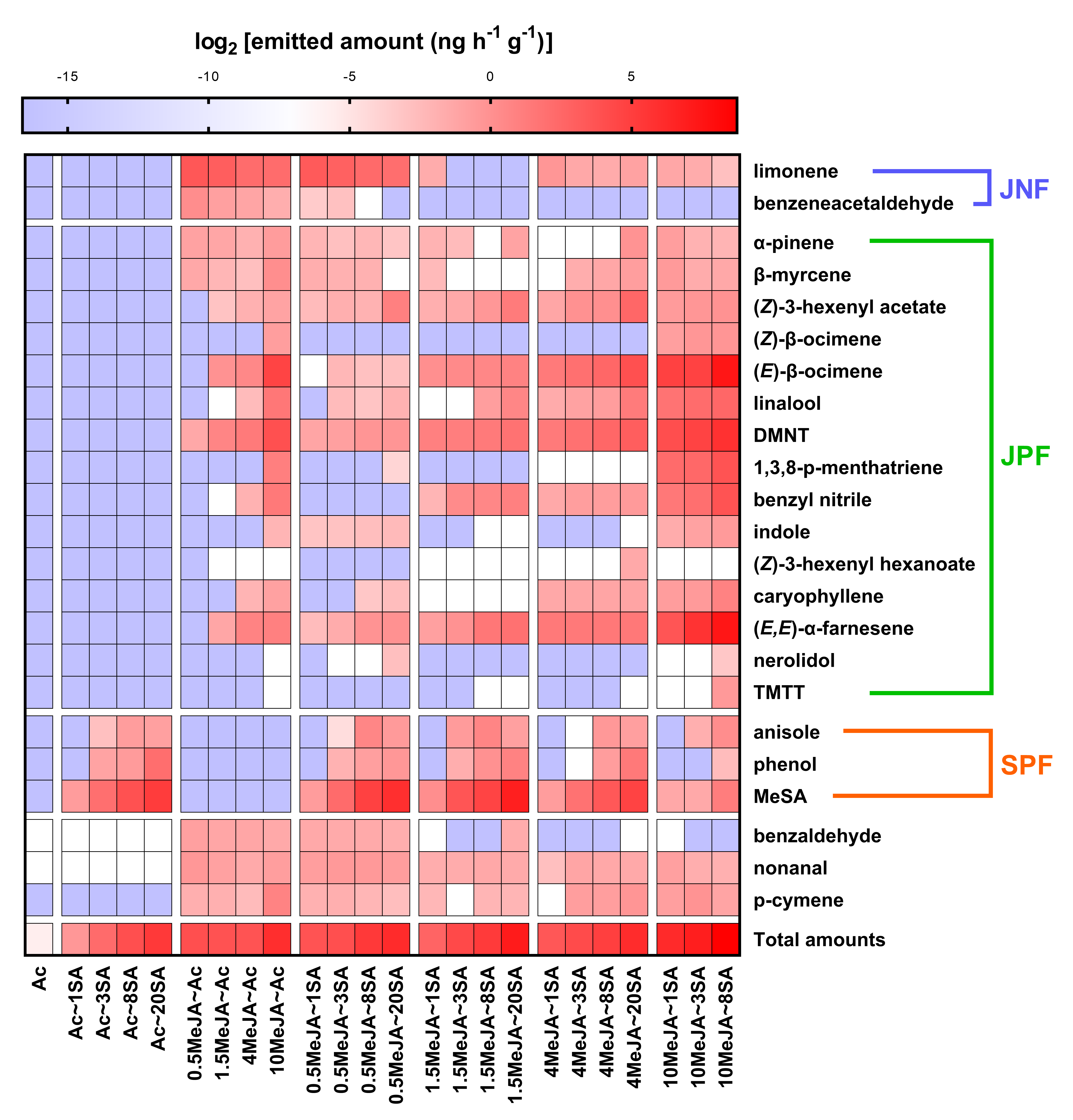


**Figure S4.** Tea plant volatiles induced both by methyl jasmonate and salicylic acid at different concentrations. JNF, JA negative features. JPF, JA positive features. SPF, SA positive features. DMNT, (*E*)-4,8-dimethyl-1,3,7-nonatriene. TMTT, (*E*,*E*)-4,8,12-trimethyl-1,3,7,11-tridecatetraene. MeSA, methyl salicylate. For treatment abbreviations, see Table 1. Data are given as log_2_ (mean emitted amount), *n* = 4. Blue and white cells represent, respectively, that compound was not detected or detected at less than 0.01 ng h^-1^ g^-1^ (signal to noise ratio = 3).

**Table S1. Studies (from 2000 to 2021) on effects of jasmonic acid (JA)–salicylic acid (SA) interactions in various plant species with affected downstream metabolites and ecological outcomes.**

| **Plant species** | **Elicited sequence of JA and SA pathways** | **Concentration of JA pathway elicitors (mM)** | **Concentration of SA pathway elicitors (mM)** | **Effects of SA on JA pathway** | **Effects of JA on SA pathway** | **References** |
| --- | --- | --- | --- | --- | --- | --- |
| *Solanum lycopersicum* | Simultaneous | 1.2 | 1.5 | Antagonism | - | 33 |
|  | Simultaneous | 0.3 | 0.5 | Antagonism | - |  |
|  | Pre-JA and post-SA at 2 d intervals | 1.2 | 1.5 | Antagonism | - |  |
|  | Pre-JA and post-SA at 2 d intervals | 0.3 | 0.5 | None | None |  |
|  | Pre-SA and post-JA at 2 d intervals | 1.2 | 1.5 | Antagonism | - |  |
|  | Pre-SA and post-JA at 2 d intervals | 0.3 | 0.5 | Antagonism | - |  |
|  | Pre-JA and post-SA at 24 h intervals | 0.1 | 0.06 | Synergism | Synergism | 47 |
|  | Simultaneous | 1.2 | 1.5 | Antagonism | - | 34 |
| *Cucumis sativus* | Simultaneous | 0.5 | 0.25 | - | Antagonism | 35 |
| *Pisum sativum* | Simultaneous | 0.01 | 0.01 | Antagonism | - | 37 |
| *Arabidopsis thaliana* | Simultaneous | 1 | 0.1 | Antagonism | Antagonism | 38 |
|  | Pre-SA and post-JA at 1 h intervals | 0.5 | 0.45 | Antagonism | - | 45 |
|  | Simultaneous | 0.01 | 0.05 | Antagonism | - | 40 |
|  | Pre-JA and post-SA at 24 h intervals | 0.1 | 0.06 | Synergism | Synergism | 47 |
| *Zea mays* | Pre-SA and post-JA at 15 h intervals | 0.005 | 0.1 | None | - | 46 |
|  | Pre-SA and post-JA at 15 h intervals | 0.05 | 0.1 | Synergism | - |  |
|  | Pre-SA and post-JA at 15 h intervals | 0.5 | 0.1 | Antagonism | - |  |
| *Fragaria* × *ananassa* | Simultaneous | 1 | 0.008 | Antagonism | - | 39 |
|  | Simultaneous | 2 | 0.008 | Antagonism | - |  |
|  | Simultaneous | 1 | 0.016 | Antagonism | - |  |
|  | Simultaneous | 2 | 0.016 | Antagonism | - |  |
| *Plantago lanceolata* | Simultaneous | 0.25 | 0.25 | Antagonism | - | 7 |
|  | Simultaneous | 0.5 | 0.5 | Antagonism | Antagonism |  |
| *Phaseolus lunatus* | Simultaneous | 0.001 | 1 | Antagonism | - | 11 |
|  | Simultaneous | 0.01 | 1 | Antagonism | - |  |
|  | Simultaneous | 0.1 | 1 | Antagonism | - |  |
|  | Simultaneous | 1 | 1 | Antagonism | - |  |
|  | Pre-SA and post-JA at 24 h intervals | 1 | 0.001 | - | Antagonism |  |
|  | Pre-SA and post-JA at 24 h intervals | 1 | 0.01 | - | Antagonism |  |
|  | Pre-SA and post-JA at 24 h intervals | 1 | 0.1 | - | Antagonism |  |
|  | Pre-SA and post-JA at 24 h intervals | 1 | 1 | - | Antagonism |  |
|  | Pre-JA and post-SA at 24 h intervals | 0.001 | 1 | Antagonism | - |  |
| *Nicotiana tabacum* | Pre-JA and post-SA at 24 h intervals | 0.1 | 0.06 | Synergism | Synergism | 47 |
| *Capsicum annuum* | Pre-JA and post-SA at 24 h intervals | 0.1 | 0.06 | Synergism | Synergism | 47 |
|  | Simultaneous | 0.25 | 0.05 | Antagonism | Antagonism | 44 |
| *Thevetia peruviana* | Simultaneous | 0.3 | 0.003 | Antagonism | - | 43 |
| *Vitis vinifera* | Simultaneous | 0.2, 1, 5, 25 | 0.04, 0.125, 1, 5 | Antagonism | - | 36 |
| *Chlamydomonas reinhardtii* | Simultaneous | 1 | 0.5 | Antagonism | Antagonism | 41 |
| *Marchantia polymorpha* | Simultaneous | 0.5 | 0.001 | - | Antagonism | 42 |

“-” indicates not investigated. Papers were screened from a review^5^ and Web of Science Core Collection database with publication dates from 2000-01-01 to 2021-12-31. Query used for Advanced Search Query Builder in Web of Science^TM^ was “((TI=(jasmonate and salicylate) OR TI=(jasmonate and salicylic) OR TI=(jasmonic and salicylic) OR TI=(jasmonic and salicylate)) AND (DT==("ARTICLE") AND SILOID==("WOS"))”.
